# Generating new mixtures of food additives with antimicrobial and cytotoxic potency against *Bacillus cereus* and *Staphylococcus aureus*

**DOI:** 10.1002/fsn3.2691

**Published:** 2022-01-04

**Authors:** Samy Selim, Mohammed S. Almuhayawi, Mohammed H. Alruhaili, Shadi A. Zakai, Mona Warrad

**Affiliations:** ^1^ Department of Clinical Laboratory Sciences College of Applied Medical Sciences Jouf University Sakaka Saudi Arabia; ^2^ Department of Medical Microbiology and Parasitology Faculty of Medicine King Abdulaziz University Jeddah Saudi Arabia; ^3^ Department of Clinical Laboratory Sciences College of Applied Medical Sciences at Al‐Qurayyat Jouf University Al‐Qurayyat Saudi Arabia

**Keywords:** antimicrobial activity, cytotoxic activity, nisin, phloxine B, sorbic acid, synergistic effect

## Abstract

Food additives have been shown to help regulate or prevent the spread of microbes during food manufacturing. Phloxine B, nisin, and sorbic acid were tested to see whether they had a synergistic impact on the inactivation of *Bacillus cereus* and *Staphylococcus aureus*, respectively. The combination of phloxine B and nisin had a synergistic interaction (FICI: 0.25–0.50) against *B. cereus*, where it demonstrated an additive effect among the three combinations examined (FICI: 0.91). A time‐kill test was used in both cases to verify that a food additive combination has synergistic antibacterial action against *B. cereus* and *S. aureus*. *B. cereus* had a 50% reduction in bacterial colony count after 10 h, whereas *S. aureus* had a 60% reduction after 6 h of their independent impacts after 48 h. Phloxine B, nisin, and sorbic acid demonstrated synergistic antibacterial action and might be used as a source of safe and potent antibacterial agents in the pharmaceutical and food industries.

## Graphical abstract

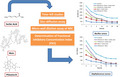

## 1 INTRODUCTION

Anything that is not habitually considered or utilized as food and is added to or used in or on foods at any stage might be called an addition if it commits to changing food value, smoothness, stability, flavor color, alkalinity, or sourness in a positive way. The term "processing aids" refers to anything utilized in or added to food at any point during the production process, including when the meal is being served (Food and Drug Administration (FDA), 2007, Hallas‐Møller et al., 2020). Additionally, food additives may have a role in limiting the spread of bacteria or perhaps preventing it altogether. Only a limited number of chemical compounds can be used in food and pharmaceutical preservatives to protect against real or prospective toxicity to end users (Conner, 1993; Hugo & Russell, 1991).

A bactericidal effect on *S. aureus* has been observed with the dye phloxine B, which is a 4,5,6,7‐tetrachlorofluorescein disodium salt. However, phloxine B is only effective against gram‐positive bacteria, and its antimicrobial activity is light‐dependent. Phloxine B batches certified by the FDA are allowed in the USA for consumption in dyeing cosmetics and swallowed drugs with an adequate regular intake for humans set by the FDA at 1.25 mg/kg of body weight (European Commission Regulation, 2011; Federal Register, 1982). The growth of bacteria was completely halted when dye was added at concentrations of 50 or 100 µg/ml. However, the widespread use of phloxine B as a color additive in foods, drugs, and cosmetics raises the possibility that bacteria will be exposed to small amounts of it over time, leading to the development of resistance to its antimicrobial activity (Rasooly & Weisz, 2002; Rasooly, 2005).

Food preservative nisin (E 234), a peptide antibiotic created by *Lactococcus lactis*, is widely used and has high antimicrobial activity against many different gram‐positive bacteria. The FAO/WHO appropriate nisin as a safe food additive in 1969. Since then, it has been widely accepted as a safe food additive. Nisin is a natural biopreservative approved for use in more than 50 countries and has had a significant impact on the food industry (de Arauz et al., 2009). In the United States, the Food and Drug Administration (FDA) (2007) approved nisin in Managing Food Safety, and designated and generally recognized it as harmless for consumption in administered cheeses (Cotter et al., 2005). Additionally, nisin is effective at preventing the spread of spores from the bacteria *Clostridium* and *Bacillus* (Rayman et al., 1981). In addition to nisin activity against gram‐positive organisms, studies have shown that nisin is also active against gram‐negative organisms, albeit at a lower concentration and usually in conjunction with chelating agents (Abee et al., 1994; Delves‐Broughton et al., 1996). The nanomolar activity of nisin and its lack of human toxicity have made it a widely accepted food additive for controlling food spoilage around the world (Thomas et al., 2000). Nisin caused cytoplasmic leakage from treated samples, despite the fact that it had multiple abnormalities. Nisin inhibits cell wall formation near the partition site, where peptidoglycan construction is expedited, as the primary site of cell division. Nisin also disrupts the regulation of cell envelope formation, resulting in atypical cell growth (Hyde et al., 2006).

Sorbic acid or sorbitol (2,4‐hexadienoic acid) is a paraben commonly used in the food and pharmaceutical industries. Sorbic acid, which was originally derived from rowanberry, can now be found in a wide range of plants (Kallscheuer, 2018), but it is still manufactured synthetically for commercial use. It is possible to preserve pharmaceutical or food products that contain a lot of water by using these compounds because of the chemical or physical interactions between them and the water. Potassium sorbate and sorbic acid are now widely used in the pharmaceutical industry for antimicrobial preservation in concentrations between 0.1 and 0.2 percent (Nemes et al., 2020). There is general consensus that they are safe for human consumption, despite a paucity of toxicology and biocompatibility data. Sorbate's antimicrobial action is not fully understood, but it is thought to be primarily established on microbes' intracellular acidification (Bagar et al., 2009; Plumridge et al., 2004). The weak carboxylic acid penetrates the cell membrane and releases a proton, acidifying the cytosol and disrupting catabolic pathways as a result (Mira et al., 2010). Antimicrobial action of sorbates declines as extracellular pH rises because only the nonionized form can enter cells, according to the research (Wang et al., 2018). Because of this, the formation of bacterial biofilms can be reduced with the use of sorbates (Sullivan et al., 2020). These prior studies prompted us to investigate the antimicrobial synergistic properties of some food additives (phloxine B, nisin, and sorbic acid) against two common pathogens, *B. cereus* and *S. aureus*. In addition, cytotoxic activity on *B. cereus* and *S. aureus* uses the time‐kill method. These previous researches compelled us to study the antibacterial synergistic capabilities of food additives (phloxine B, nisin, and sorbic acid) against two prevalent pathogens, *B. cereus* and *S. aureus*. Additionally, the time‐kill approach was used to test for cytotoxic activity against *B. cereus* and *S. aureus*.

## 2 MATERIALS AND METHODS

### 2.1 Chemicals

All chemicals and solvents were achieved from Sigma‐Aldrich Co. Ltd.

### 2.2 Antimicrobial tests

#### 2.2.1 Microbial strains

The modified Kirby‐Bauer disk diffusion process was used to conduct the agar diffusion assay (Selim et al., 2013). 0.9 percent NaCl solution contained one loop of each of the test organisms (10 isolates of *B. cereus* and 10 isolates of *S. aureus*). Microorganism strains from Microbiological Laboratory Collection Library, Clinical Laboratory Sciences, College of Applied Medical Sciences, Jouf University, were utilized in this investigation. A sterile petri dish was filled with nutrient agar for bacterial strains that had been inoculated with the corresponding organism's suspension.

#### 2.2.2 Disk‐diffusion assay

**FIGURE 1:**
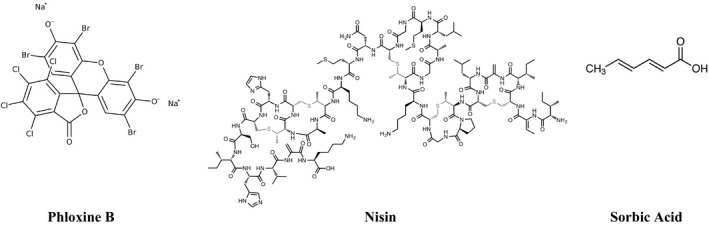
Chemical structure of selected studied food additives

The food additives (phloxine B, nisin, and sorbic acid; Figure 1) were produced in dimethyl sulfoxide (DMSO) and sterilized with 0.45‐m Millipore filters at a final concentration of 50 µg/ml. The disk diffusion technique was used to put 10^8^ cfu/ml of bacteria on Mueller‐Hinton agar (MHA) to test for antimicrobial susceptibility (Selim, 2011). Five micrograms/disk was applied to each of the 6‐mm disks, which were then placed on top of the inoculated agar. DMSO was used to prepare the negative controls. One strain/isolate of each microorganism tested was compared with positive reference standards such as amoxicillin (30 micrograms/disk), gentamicin (30 micrograms/disk), and streptomycin (30 micrograms/disk). For clinical bacterial strains, the inoculated plates were incubated for 24 h at 37°C. The zone of inhibition against the test organisms was used to assess antimicrobial activity.

#### 2.2.3 Micro‐well dilution assay of MIC

Minimal inhibitory concentration (MIC) values were determined using 12‐h broth cultures, the bacterial strains' inocula were made, and the suspensions were adjusted to 0.5 McFarland turbidity standard. The additives were first dissolved in 10% DMSO and then diluted to the highest concentration (5 µg/ml) to be tested, and then, serial twofold dilutions were made in a concentration range from 0.1 to 5 µg/ml in 10‐ml sterile test tubes containing nutrient broth. The additives' MIC values against microbial strain isolates were determined using a micro‐well dilution method. As an overview, 95 microliters of the growth medium (nutrient broth) was added to each well, along with 5 microliters of the inocula. In the first wells, 100 L aliquot of the initial 50 µg/ml of stock solutions of the compounds was added to start things off. Next, 100 µl of each of their serial dilutions was added to six different wells in the plate. This well served as a negative control and contained 195 microliters of nutrient broth without compound and 5 microliters of inocula on each test strip. Each well had a total volume of 200 µl at the end of the experiment. A sterile plate sealer was used to protect the plate from contamination. Each well's contents were mixed for 20 s on a plate shaker at 300 rpm before being incubated for 24 h at the appropriate temperature. Samples from clear wells were plated on nutrient agar medium in order to determine microbial growth.

#### 2.2.4 Time‐kill studies

For time‐kill studies, an inoculum of 5 × 10^8^ cfu/ml^−1^ was prepared for each isolate by dilution of an actively growing culture in nutrient broth with the inoculum used for each isolate verified by a total viable count, as previously described for each isolate. One milliliter samples of the initial inoculum were mixed with either additive solution and Tween 80 (for testing purposes) or Tween 80 alone (for the control). Samples (100 µl) were taken in triplicate at different times, and serial tenfold dilutions were made and plated on nutrient agar in McCartney bottles for all isolates that were shaken (100 rpm) at 37°C (Oxoid). After an overnight incubation at 37‐degree Celsius, the total number of viable cells was calculated.

### 2.3 Determination of fractional inhibitory concentration index

Through the use of the checkerboard titration method, the inhibitory concentration index fraction was calculated. To do this, the combined MICs of active additives were estimated using the microbroth dilution method (Clinical & Laboratory Standards Institute, 2005) after their separate MICs were obtained. Selective broth media supplied to the micro‐titer plates, along with 10 μl of the working inoculum (5 × 10^5^ cfu/ml). 100 μl of various concentrations of test food additives (1:1 v/v) ranging from 1/32 MIC to 4 MIC was applied to the wells. The growing parameters used to determine the individual MIC remained the same. Then, the fractional inhibitory concentration (FIC) index was intended for each mixture of food additive to estimate synergistic activities (Karthikeyan et al., 2015; Meyer et al., 1982; Xia et al., 2010). MICs of the individual food additive in each arrangement were converted into FICs as monitored by Xia et al. (2010):

$$\text{FIC}\;\text{Food}\;\text{additive}=\frac{\text{MIC}\;\text{Food}\;\text{additives}\;\text{in}\;\text{mixtures}}{\text{MIC}\;\text{Food}\;\text{additive}\;\text{alone}}$$

Fractional inhibitory concentration index (∑FIC) was calculated using the standard plan as designated by Xia et al. (2010) and Karthikeyan et al. (2015):

$$\sum_{k=1}^{n}\left(\text{FIC}\right)$$

Synergistic actions were distinct as ∑FIC ≤0.5, additive was defined as ∑FIC >0.5 to <2, and antagonism was defined as ∑FIC ≥2 (Leclercq et al., 1991; Xia et al., 2010). The formula used to determine fractional inhibitory concentration index (FICIs) is as follows:

$$\text{FICI}=\frac{\text{MIC}\;\text{of}\;\text{AD}1\;\text{in}\;\text{combination}\;\text{with}\;\text{AD2}}{\text{MIC}\;\text{of}\;\text{AD1}\;\text{alone}}+\frac{\text{MIC}\;\text{of}\;\text{AD2}\;\text{in}\;\text{combination}\;\text{with}\;\text{AD}1}{\text{MIC}\;\text{of}\;\text{AD2}\;\text{alone}}$$

where AD_1_ and AD_2_ are tested two different food additives. All the trials were repeated thrice.

### 2.4 Statistical analysis

The data were exposed to one‐way analysis of variance for means of comparison and significant differences according to Duncan's multiple range test. SPSS (version 11.0) was used to achieve the statistical investigation.

## 3 RESULTS AND DISCUSSION

Antimicrobial characteristics have long been identified in food additives, which can include an extensive range of different ingredients. When used alone in vitro, they demonstrate potential antibacterial efficacy against a wide range of microorganisms, including food‐borne pathogens and degeneration. However, the combined antibacterial actions of these drugs appear to be rare. For the most part, antimicrobial medication combinations have shown to be a critical characteristic since they improve efficacy‐using compounds with synergistic or additive action, prevent drug resistance, and reduce necessary dosages while also decreasing cost and adverse/toxic side effects (Bag & Chattopadhyay, 2015).

**FIGURE 2:**
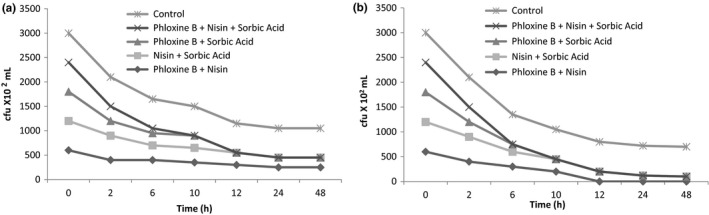
Time kill curves of (a) *Bacillus cereus* and (b) *Staphylococcus aureus* by selected additive mixtures

The food additives (phloxine B, nisin, and sorbic acid) investigated for their inhibitory (antibacterial) effects on two bacterial species (*B. cereus* and *S. aureus*). To test for antibacterial activity against food spoilage bacteria *B. cereus* and *S. aureus*, the concentration of 50 µg/ml produced encouraging results (inhibition zone diameter 11 mm) (Table 1). The synergistic antibacterial activity of these three active food additives was then investigated using an antimicrobial combination study. In an antibacterial combination research, only the phloxine B and nisin combination exhibited synergistic interaction (FICI: 0.25–0.50) against *B. cereus* where it showed additive impact among the three studied combinations (FICI: 0.91). Various other combinations (FICI: 0.55–1.37) were shown to have an additive impact on all of the microorganisms that were examined. There was no negative outcome (Table 2). A time‐kill test was used to verify the synergistic antibacterial activity of a food additive combination. When compared to the original bacterial colony count (*B. cereus* in 10 h and *S. aureus* in 6 h) of their separate effects after 48 h, the combination of phloxine B + nisin + sorbic acid reduced the bacterial colony count by >2 log_10_ (Figure 2). These findings corroborated the prior experiment's discovery of synergistic antibacterial action of phloxine B + nisin + sorbic acid.

**TABLE 1:** Inhibition zone diameter and MIC of food additives against *Bacillus cereus* and *Staphylococcus aureus* using agar well diffusion assay

Food additives	*B. cereus*				*S. aureus*			
	Inhibition zone diameter^a^	MIC (µg/ml)			Inhibition zone diameter^a^	MIC (µg/ml)		
		MIC	MIC_50_	MIC_90_		MIC	MIC_50_	MIC_90_
Phloxine B	17 ± 1.20	20	100	200	19 ± 1.05	10	10	50
Nisin	11 ± 1.31	320	160	320	14 ± 1.00	200	250	300
Sorbic acid	13 ± 1.09	20	100	160	15 ± 1.09	320	350	1000
DMSO (negative control)	—	—	—	—	—	—	—	—

Note

Concentration of 50 µg/ml of the additives was prepared in dimethyl sulfoxide (DMSO). Results are mean ± SD of triplicate experiments.

^a^ Sensitive (inhibition zone diameter ≥11 mm: Bauer et al., 1966).

**TABLE 2:** Results of checkerboard titration technique's synergistic effects on food‐borne microorganisms versus additive mixtures

Food additive mixtures	*Bacillus cereus*				*Staphylococcus aureus*			
	No. of synergistic isolates	FIC^a^	FICI	Remarks^b^	No. of synergistic isolates	FIC	FICI	Remarks^b^
Phloxine B (a) + nisin (b)	7	0.63 (a)	0.49	S	9	0.92 (a)	0.91	Add
		0.35 (b)				0.89 (b)		
Nisin (b) + sorbic acid (c)	5	0.56 (b)	0.55	Add	8	0.88 (b)	0.80	Add
		0.55 (C)				0.97 (C)		
Phloxine B (a) + sorbic acid (c)	7	0.78 (a)	0.78	Add	9	1.12 (a)	1.06	Add
		0.77 (b)				1.00 (b)		
Phloxine B (a) + nisin (b) + sorbic acid (c)	8	1.15 (a)	0.99	Add	10	1.98 (a)	1.37	Add
		0.85 (b)				0.86 (b)		
		0.98 (c)				1.26 (c)		

^a^ The fractional inhibitory concentration (FIC) index was calculated as the sum of the FICs for each individual food additive.

^b^ ∑FIC ≤0.5 was defined as synergism, and ∑FIC >0.5 to <2 was defined as additive.

Several studies have shown that chemical growth control of food deterioration and food‐borne pathogens is effective. In order to compare the known effects of antimicrobial with the unknown effects of food additives, a database based on antibiotic susceptibility on the investigated bacterial species is used. Microorganism multiplication can be slowed or stopped using food additives. There are a finite number of chemical substances that can be employed as preservatives in food and pharmaceuticals (Branen, 1983). The usage of food additives has exploded in the last 30 years, reaching a total annual volume of over 200,000 tons. For the most part, chemical preservatives have been employed to resist certain bacteria and can also improve or contribute to the flavor of acidified or fermented foods.

As a food preservative, phloxine B was shown to be the most efficient addition against the tested bacterial species. Phloxine B had an antibacterial activity against both gram‐positive and gram‐negative bacteria, which was in agreement with that of Rasooly (2005) and Federal Register (1982). After adding 50–100 µg/ml of phloxine B to the growing medium, the bacterial growth ceased completely (Rasooly & Weisz, 2002). Against gram‐positive and gram‐negative bacteria, nisin has antibacterial action (Abee et al., 1994; Delves‐Broughton, 1996). Nisin has been shown to help reduce spoilage microorganisms in dairy, seafood, juice, and vegetables. Foods such as meat, liquids, and baked goods can benefit from the use of organic acid to help limit the growth of germs and molds. Organic acids are often used throughout a wide range of industries, including food production, processing, and manufacturing.

## 4 CONCLUSION

Thus, a combination of phloxine B, nisin, and sorbic acid showed synergistic antibacterial action and might be utilized as a source of nontoxic and powerful antibacterial agents in the pharmaceutical and food sectors. By working together, they may be more effective against bacteria at low concentrations, reducing their unwanted side effects and making them more readily used in food preservation systems. More research on their use in food components and mechanisms of action is required to improve their practical application in the food system. It is possible that this research will serve as a springboard for more in‐depth investigations in the future. Food additive synergy with antibacterial activity is a relatively new concept, and this study is the first to examine it.

## CONFLICT OF INTEREST

The authors declare that there is no conflict of interests.

## AUTHOR CONTRIBUTIONS

**Samy Selim:** Conceptualization (equal); Data curation (equal); Formal analysis (equal); Funding acquisition (equal); Investigation (equal); Methodology (equal); Project administration (equal); Resources (equal); Software (equal); Supervision (equal); Validation (equal); Visualization (equal); Writing – original draft (equal); Writing – review & editing (equal). **Mohamed almuhayawi:** Conceptualization (equal); Data curation (equal); Formal analysis (equal); Funding acquisition (equal); Investigation (equal); Methodology (equal); Project administration (equal); Resources (equal); Software (equal); Supervision (equal); Validation (equal); Visualization (equal); Writing – original draft (equal); Writing – review & editing (equal). **Mohammed H. Alruhaili:** Conceptualization (equal); Data curation (equal); Formal analysis (equal); Funding acquisition (equal); Investigation (equal); Methodology (equal); Project administration (equal). **Shadi Ahmed Zakai:** Conceptualization (equal); Data curation (equal); Formal analysis (equal); Writing – review & editing (equal). **Mona Warrad:** Conceptualization (equal); Data curation (equal); Formal analysis (equal); Funding acquisition (equal); Investigation (equal); Methodology (equal); Project administration (equal); Supervision (equal); Validation (equal); Visualization (equal); Writing – original draft (equal); Writing – review & editing (equal).

## ETHICAL APPROVAL

This article does not contain any studies with participants or animals requiring ethics approval.

## Data Availability

The datasets used or analyzed during the current study are available from the corresponding author on reasonable request.
